# Carbon Catabolite Repression in Yeast is Not Limited to Glucose

**DOI:** 10.1038/s41598-019-43032-w

**Published:** 2019-04-24

**Authors:** Kobi Simpson-Lavy, Martin Kupiec

**Affiliations:** School of Molecular Cell Biology & Biotechnology, Tel Aviv University, Ramat Aviv, 69978 Israel

**Keywords:** Metabolic engineering, Gene regulation

## Abstract

Cells adapt their gene expression and their metabolism in response to a changing environment. Glucose represses expression of genes involved in the catabolism of other carbon sources in a process known as (carbon) catabolite repression. However, the relationships between “poor” carbon sources is less characterized. Here we show that in addition to the well-characterized glucose (and galactose) repression of ADH2 (alcohol dehydrogenase 2, required for efficient utilization of ethanol as a carbon source), *ADH2* expression is also inhibited by acetate which is produced during ethanol catabolism. Thus, repressive regulation of gene expression occurs also between “poor” carbon sources. Acetate repression of *ADH2* expression is via Haa1, independently from the well-characterized mechanism of AMPK (Snf1) activation of Adr1. The response to extracellular acetate is attenuated when all three acetate transporters (Ady2, Fps1 and Jen1) are deleted, but these deletions do not affect the acetate response resulting from growth with glucose or ethanol as the carbon source. Furthermore, genetic manipulation of the ethanol catabolic pathway affects this response. Together, our results show that acetate is sensed intracellularly and that a hierarchical control of carbon sources exists even for “poor” carbon sources.

## Introduction

Cells have evolved to respond appropriately to changes to both their intracellular and extracellular environments via multiple sensing mechanisms. Some environmental signals [such as osmotic stress^1^] or nutrients [such as glucose^2^] are sensed at the plasma membrane, whereas others [such as nitrogen^3^, or reactive oxygen species^4^] are sensed intracellularly, despite the drawback that damage might be inflicted before the cell can mount its response. In many studied organisms, glucose is the preferred carbon source^5^. In yeasts^6–8^, multicellular fungi^9–11^ bacteria^12^ and metazoa^13^ the presence of glucose in the environment prevents the utilization of other available carbon sources, by mechanisms known by the general title of “catabolite repression”. In the yeast *Saccharomyces cerevisiae*, when glucose is present in the extracellular medium, uptake and catabolism of other carbon sources is repressed^6^ via three signaling pathways; inhibition of AMPK^Snf1 ^^14^, activation of PKA^15,16^, and the regulation of transporter expression and stability at the plasma membrane by the yeast casein kinases Yck1 and Yck2^17^. Despite the fact that this glucose-mediated catabolite repression has been extensively researched, whether similar hierarchies exist for other carbon sources, and how these different sources are sensed, remains unclear.

Acetate is of interest to oenologists, both as a factor affecting wine spoilage^18^ and as a source of aroma compounds, such as acetate-esters^19^. Alterations in gene expression of the ethanol-acetate pathway have been previously shown to affect acetic acid production during fermentation^20^. However, acetate is also toxic to cells, resulting in programmed cell death^21^ and a reduction in chronological life span^22^. This has led to its use as a food preservative^23^, but is of concern to the biofuel industry. Ethanol production from lignocellulose is limited by the accumulation of acetic and other weak organic acids^24–26^. A search for genes that can reduce this toxicity found that overexpression of the Haa1 transcription factor results in resistance to acetic acid and increased ethanol yield, by mediating increased expression of target genes^27^. Indeed, Haa1 is responsible for the activation of ~80% of the genes that respond to acetic acid^28^, despite the fact that only about half of these genes have the Haa1 binding site in their promoters^29^.

Acetate is actively transported into the cell through the main transporters Jen1 and Ady2^30,31^, which are subject to strong glucose repression and inhibition of activity^17,30,31^. In addition, the undissociated acid undergoes passive/facilitated diffusion through the Fps1 aquaglyceroporin^32,33^. Therefore, in glucose-containing media Fps1 is the only route by which acetic acid enters the cell, and deletion of Fps1 results in acetic acid resistance. Although Ady2 is the major importer of acetate, deletion of *ADY2* does not affect the response to acetate^30^.

In addition to being an environmental resource, acetate is also a metabolic product formed during fermentation and ethanol catabolism in a pathway conserved across eukaryotes. There are five *ADH* genes in the *S. cerevisiae* genome. Cells expressing only *ADH1* produce ethanol during fermentation of glucose comparably to wild-type cells, and can metabolise ethanol^34^ Adh2 has a 20-fold higher affinity for ethanol than Adh1^35^ and thus cells expressing only *ADH2* do not produce ethanol during glucose fermentation, but are capable of ethanol catabolism^34^. The mitochondrial Adh3 is expressed at low levels and forms the mitochondrial component of the ethanol-acetyladehyde shuttle to regenerate mitochondrial NAD from NADH^36^. However, cells expressing only *ADH3* are capable to both produce and utilize ethanol (similarly to *ADH1*). Cells expressing solely *ADH4* or *ADH5* are incapable of producing or metabolizing ethanol^34^. Acetylaldehyde is oxidized to acetic acid primarily by the cytoplasmic Ald6 and the mitochondrial Ald4 and Ald5 enzymes; the first two use NADP as a co-factor, whereas the latter utilizes NAD^37,38^. Acetate is then conjugated to coenzyme-A in the cytoplasm by two acetyl-coA synthetases: Acs1, which is glucose-repressed^39^ and Acs2, which is constitutively expressed^40^. Cytoplasmic Acetyl-coA has important roles in fatty acid synthesis^41^, acetylation of proteins such as histones^42^, biosynthesis of sterols and amino acids, and for entry into the Krebs’ cycle via the glyoxylate pathway, which is repressed in the presence of glucose^43^. A simplified diagram of metabolic pathways pertaining to ethanol and acetate metabolism is presented in Fig. S1.

Despite the central role played by acetate in yeast metabolism, the location of acetic acid sensing has not been yet determined. Here we demonstrate that acetate, a product of both fermentation and ethanol catabolism and itself a carbon source, inhibits expression of genes involved in ethanol catabolism. We show that acetate is sensed intracellularly, and a consequence of this is that ethanol metabolism results in the induction of the acetate response. Our results uncover the existence of catabolite repression among sugars that are considered “poor” carbon sources.

## Results

### Acetate represses expression of genes involved in ethanol metabolism

**Figure 1:**
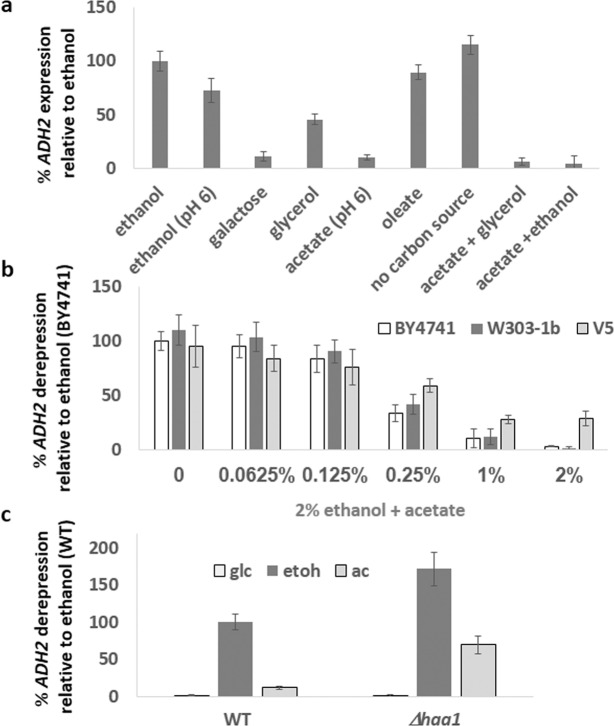
Cells were grown overnight in 4% glucose. In the morning, cells were diluted and grown for an additional 3 hours in 4% glucose media. After measuring the basal *ADH2* expression (t = 0), cells were washed three times with DDW and resuspended in media containing the indicated carbon source(s). *ADH2* expression was determined after 3 hours. Rate of expression is normalized against WT cells (BY4741a for figure **b**) in ethanol media. N = 3. Error bars are one standard deviation.

Expression of the *ADH2* gene, encoding alcohol dehydrogenase, is tightly regulated by carbon source, being low in the presence of glucose, and high on poor carbon sources. Although the inhibition of *ADH2* expression by glucose via the inactivation of Snf1 has been well-characterized (Snf1 is needed to activate the Adr1 and Cat8 transcription factors)^44^, it is still unclear whether *ADH2* expression requires a positive signal from the poor carbon source. We therefore examined *ADH2* expression in response to a range of carbon sources, by measuring an increase in β-galactosidase activity following transfer of cells bearing an *prADH2*::LacZ reporter from 4% glucose medium to media containing other carbon sources. As previously shown^45^, *ADH2* was repressed on medium containing glucose. Whereas earlier work demonstrated that abolition of glucose repression results in constitutive *ADH2* expression^46^, and that *ADH2* is induced by a variety of carbon sources including those whose metabolism does not involve Adh2^47^, we observed that even in the absence of any carbon source *ADH2* is highly expressed (Fig. 1a), suggesting a lack of any positive signaling mechanism. However, we consistently observed a repression of *ADH2* expression by 2% acetate (pH 6). *ADH2* expression in acetate was very low, and acetate repressed *ADH2* expression in the presence of glycerol or ethanol (Fig. 1a). Even prolonged growth of yeast (24 h) in medium containing acetate (pH6) did not result in medium acidification, suggesting that the observed effects of acetate are not due to the acidity itself, but rather by the acetate moiety. It is possible, however, that the elevated pH of this medium (pH6) results in the abrogation of *ADH2* expression. However, although *ADH2* expression is slightly attenuated when cells are grown in phosphate buffered medium (pH6) containing 2% ethanol, the acetate repression of *ADH2* expression is far more severe, indicating that this is not due to pH. Oleic acid catabolism via beta-oxidation proceeds directly to acetyl-coA and does not produce acetate. Oleic acid (0.14%) did not cause repression of ADH2 expression, suggesting that this repression is specific to acetate and not to acetyl-coA or to other poor carbon sources.

We examined the level of acetate-repression of *ADH2* expression upon transfer of cells from glucose to ethanol containing different concentrations of acetate. *ADH2* expression started to be reduced at 0.25% acetate, with more severe repression at higher concentrations (Fig. 1b). Since different genetic backgrounds of *S. cerevisiae* contain different mutations (in the “wild-type”) and this may affect respiration^48^, we examined the effects of increasing acetate concentrations on *ADH2* expression additionally in the W303-1b background (*HAP1*) and in the V5 champagne yeast background^38^. W303-1b was indistinguishable from BY4741. *ADH2* repression by acetate was slightly reduced in the V5 background, but a marked lowering of *ADH2* expression still occurred (Fig. 1b). This suggest that the effects of acetate upon *ADH2* expression is not limited to the BY4741 yeast background.

The transcription factor Haa1 mediates the response to acetate stress in glucose-grown cells; it is responsible for the increased expression of genes in the acetate response, such as *YRO2* and *TPO3*^28,29,49^. Inspection of the *ADH2* promoter showed that a potential Haa1 binding site is found at −343 before the ORF’s ATG. We found that deletion of *HAA1* dramatically enhances *ADH2* expression in ethanol and derepresses *ADH2* expression in acetate (Fig. 1c).

### Hrr25 regulates *ADH2* expression

**Figure 2:**
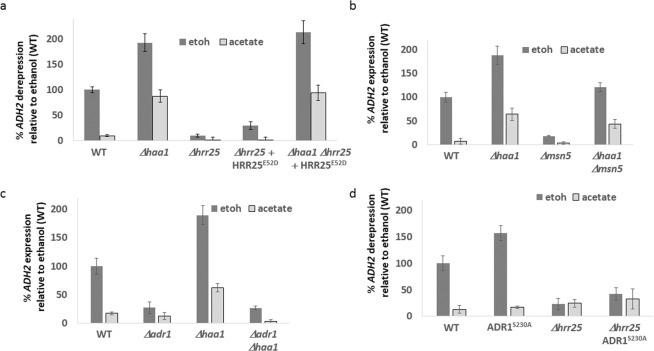
Cells were grown overnight in 4% glucose media. In the morning, cells were diluted and grown for an additional 3 hours in 4% glucose. After measuring the basal *ADH2* expression (t = 0), cells were washed three times with DDW and resuspended in media containing either ethanol (2%) or sodium acetate pH6 (2%). *ADH2* expression was determined after 3 hours. Rate of expression is normalized against WT cells in ethanol media. N = 3. Error bars are one standard deviation.

Phosphorylated Haa1 is exported from the nucleus in an Msn5 dependent manner^50^, and recently the casein kinase Hrr25 has been shown to phosphorylate Haa1 and inhibit its activity, with deletion of *HRR25* resulting in increased expression of the acetate-induced gene *YRO2* and retention of Haa1 in the nucleus even in the absence of acetate^51^. We examined *ADH2* expression in *hrr25* strains. Expression of *ADH2* was attenuated in ethanol in both *Δhrr25* and *hrr25-E52D* mutants^51^. This effect was due to Haa1 hyperactivation, as deletion of *HAA1* restored *ADH2* expression (Fig. 2a). To demonstrate the centrality of Haa1 we deleted the karyopherin-encoding gene *MSN5*. This results in an increased nuclear retention of Haa1^50^, and, as expected, in a reduction in the expression of *ADH2* in ethanol. Again, this repression could be reverted by deletion of *HAA1* (Fig. 2b).

The most studied mechanism of *ADH2* expression regulation is AMPK-Snf1 activation of the Adr1 transcription factor^44^. However, deletion of *HAA1* did not compensate for lack of Adr1 (Fig. 2c), nor did hyperactive Adr1^S230A ^^44^ restore *ADH2* expression in acetate media, or in *Δhrr25* cells in ethanol media (Fig. 2d). We therefore conclude that the Adr1 and Haa1 pathways that regulate *ADH2* expression seem to be independent from each other.

### Location of Acetate sensing

**Figure 3:**
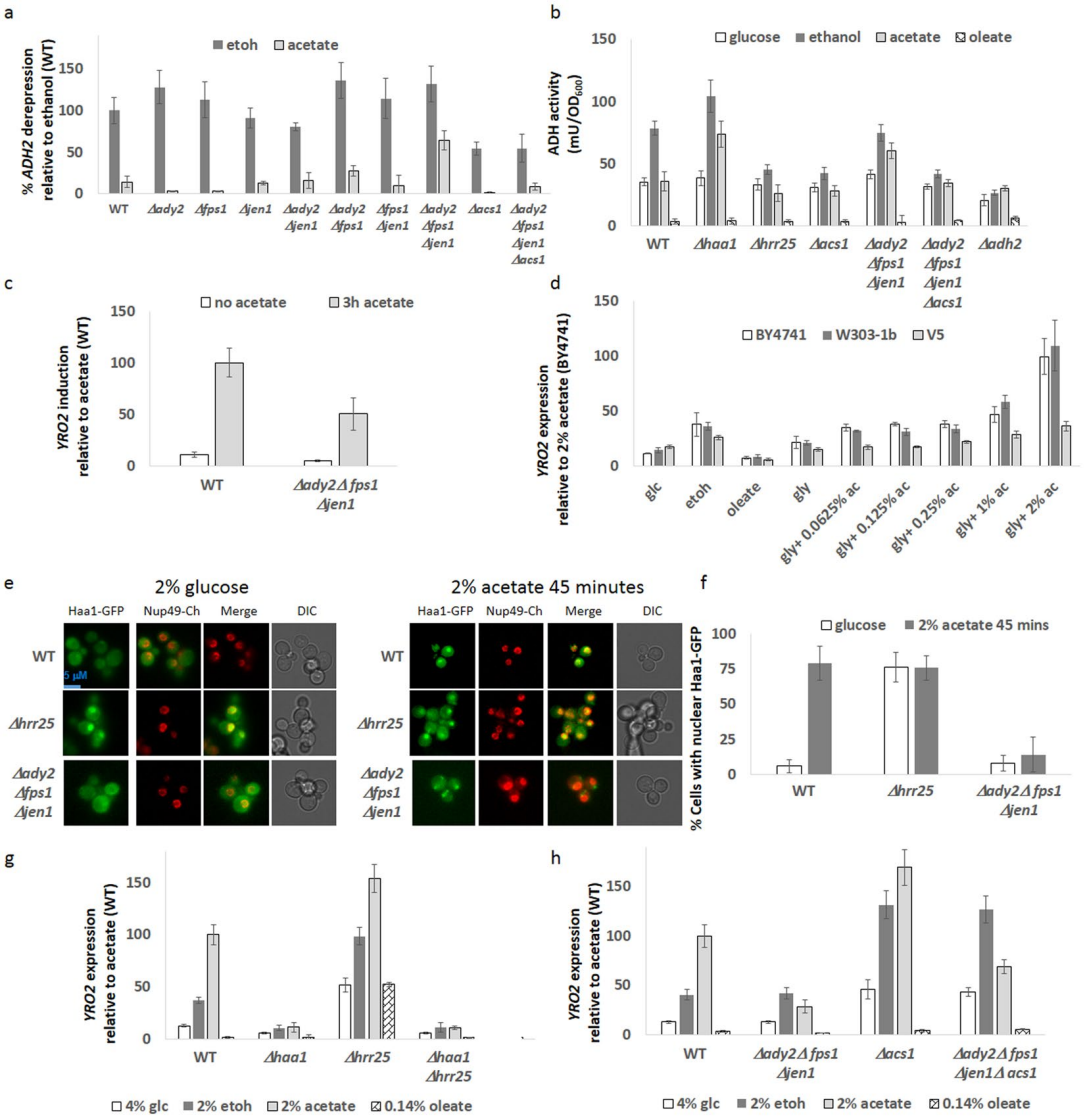
(**a**) Cells were treated as in Fig. 2 (**b**). Cells were grown overnight in the indicated media (without dilution). 0.8OD was harvested and ADH activity determined. Activity is in mU/OD_600_. N = 3. Error bars are one standard deviation. (**c**) Cells [WT or Δ*ady2*Δ*fps1*Δ*jen1*] were grown overnight in 4% glucose. In the morning, cells were diluted and grown for an additional 3 hours in 4% glucose media. After measuring basal *YRO2* expression, sodium acetate ph5.6 was added to 2% and *YRO2* expression determined after 3 hours. Rate of expression is normalized against WT cells in acetate media. N = 3. Error bars are one standard deviation. (**c**) Cells were grown for 24 hours in 5 ml of the indicated media (without dilution) and *YRO2* expression measured. Expression is normalized against BY4741 in glycerol +2% acetate medium. N = 3. Error bars are one standard deviation. (**d**) Cells were grown as in (**b**) Cells were imaged in 4% glucose media and 45 minutes following addition of acetate (pH 5.6) to 2%. (**e**) Quantification of **d** as described in Material and Methods. (**f,g**) Cells were grown for 24 hours in 5 ml of the indicated media (without dilution) and *YRO2* expression measured. Expression is normalized against WT in acetate medium. N = 3. Error bars are one standard deviation.

Anionic acetate is imported into the cell by the Jen1 and Ady2 transporters, and at low pH (below 4.76) undissociated acetic acid undergoes facilitated diffusion by Fps1 (reviewed in^21^). Acetate, being a small molecule can also passively cross the plasma membrane^32^. However, whether acetate is sensed inside or outside the cell has not been established. We generated double and triple knockouts of the acetate importers, and found that *ADH2* expression was restored to 50% of its ethanol expression levels when cells were grown in acetate media upon deletion of all three importers (Ady2, Fps1 and Jen1 - ΔΔΔ*afj*) (Fig. 3a). To confirm that this is due to acetate, we deleted *ACS1*, which converts acetate to acetyl-coA (Fig. S1) in order to lower the flux from acetate to acetyl-coA. Deletion of *ACS1* lowered *ADH2* expression and suppressed the high *ADH2* expression (in acetate) phenotype of ΔΔΔ*afj*cells (Fig. 3a). We wondered whether the changes of *ADH2* expression affect ADH activity for the oxidation of alcohols. We performed ADH assays on yeast extracts from cells grown with different carbon sources. The assay does not discriminate between different ADH enzymes, and so glucose-grown cells have an ADH activity of 20 mU/OD which is present in *Δadh2* cells (and is thus due to the other ADH enzymes, primarily Adh1). ADH enzyme activity correlates with *ADH2* gene expression above this threshold in ethanol and acetate grown cells, with *Δ*haa1 and ΔΔΔ*afj* cells having much higher ADH activity in ethanol and acetate conditions which is reversed by additional deletion of *ACS1*. Interestingly growth with oleate as the sole carbon source results in abrogation of all ADH activity, suggesting post-transcriptional regulation is occurring. Expression of *YRO2* was reduced by 50% in the ΔΔΔ*afj* strain) when acetate was added to cells for a 3 hour period (Fig. 3c). We examined the expression of *YRO2* in cells grown with differing concentrations of acetate and other carbon sources. Ethanol catabolism produces intracellular acetate, and this was sufficient to induce an acetate response (Fig. 3d). In contrast, oleic acid catabolism does not produce acetate, and *YRO2* was not expressed upon growth with oleic acid as the sole carbon source. For determining the effect of acetate concentration upon *YRO2* expression, experiments were carried out under respiratory conditions with glycerol as the carbon source, since glycerol metabolism does not involve *ADH2*. For both BY4741a and W303-1b backgrounds we observed a progressive increase in *YRO2* expression with increasing acetate concentration, though high levels of *YR02* expression occur only at 1% or higher concentrations of acetate (Fig. 3d). We note that these acetate concentrations are higher than previously reported for *YRO2* expresssion^51^ since most experiments are carried out in the presence of glucose, which causes acute acetic acid stress^21^. Similarly to *ADH2* repression (Fig. 1d), the V5 background exhibits an attenuated response to acetate.

To further confirm the importance of the acetate transporters for the acetate genetic response, we examined Haa1-GFP localization in glucose and following addition of acetate (pH 6) to 2% for 45 minutes. As previously reported, Haa1-GFP is constitutively nuclear in *Δhrr25* cells^51^. Whereas a strong nuclear Haa1-GFP localization is present in WT cells 45 minutes after addition of acetate to 2%, Haa1-GFP did not localize to the nucleus in *ΔΔΔafj* cells (Fig. 3d,e).

Since ethanol catabolism produces acetate, we reasoned that measuring *YRO2* expression (acetate-induced) in cells growing on ethanol versus acetate may provide a reasonable proxy from endogenous versus exogenous acetate. We grew cells over a 24 hour period and measured *YRO2* expression (Fig. 3g). Glucose induces about 15% of *YRO2* expression compared to acetate, presumably due to conversion of acetylaldehyde to acetate during fermentation. Likewise, ethanol induces 40% of the *YRO2* expression seen in acetate. These results imply that conversion of ethanol to acetate is faster than utilization of acetate and that acetate can function as a carbon reserve for cells. In contrast, oleic acid does not induce *YRO2* expression. Whereas *YRO2* expression was reduced by 70% in the ΔΔΔ*afj* strain during growth on acetate, expression was unaffected when cells were grown on glucose or ethanol, strongly suggesting that acetate is indeed sensed intra-celullarly. Upon deletion of *ACS1* in ΔΔΔ*afj* cells (thus restricting conversion of acetate to acetyl-coA), *YRO2* expression was somewhat increased when cells were grown on acetate (though not to the same extent as in *Δacs1* cells). Furthermore, deletion of *ACS1* also increased *YRO2* expression in glucose and ethanol grown cells, presumably due to increased acetate concentration in the cell (Fig. 3h).

### Other ethanol metabolic genes are also acetate-repressed

We analyzed expression of the other genes in the ethanol to acetyl-coA pathway (Fig. S1) upon growth on glucose, ethanol, acetate or oleic acid as the sole carbon source. Oleic acid was included as its catabolism provides an alternate source of cytoplasmic acetyl-coA, but does not induce Haa1-mediated transcriptional regulation (which responds to short chain organic acids such as acetic, propionic and lactic acids)^49^. Whereas *ALD5* expression was unchanged by deletion of *HAA1*, deletion of *HAA1* resulted in modest increases of *ALD4* and *ALD6* expression in acetate and oleic acid media, suggesting that Haa1 contributes to repression of these genes when cells can generate cytoplasmic acetyl-coA (Fig. S2a–c). *ACS1* expression was glucose-repressed as previously reported^39^ and expression in acetate was partially lowered in *Δhaa1* cells (Fig. S2d). *ACS2* expression was strongest in ethanol. No effect of Haa1 could be discerned (Fig. S2e). Thus, it seems that although Haa1 does regulate other genes in the ethanol-acetyl-coA pathway to promote a lowering of acetate levels, the major regulation that Haa1 exerts is at the *ADH2* level.

### Metabolic engineering of the acetate response

**Figure 4:**
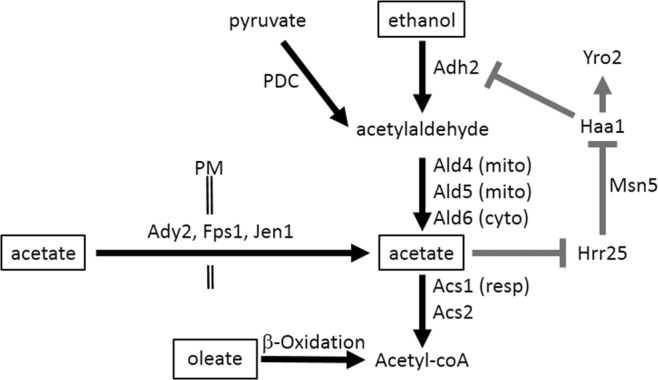
Acetate controls its own production. Model of metabolic processes and signaling pathways presented in this paper. Metabolic pathways are in black, signaling and DNA transcription in grey. Carbon sources are in boxes. PM = plasma membrane. Resp = respiration. Cyto = cytoplasm. Mito = mitochondria

**Figure 5:**
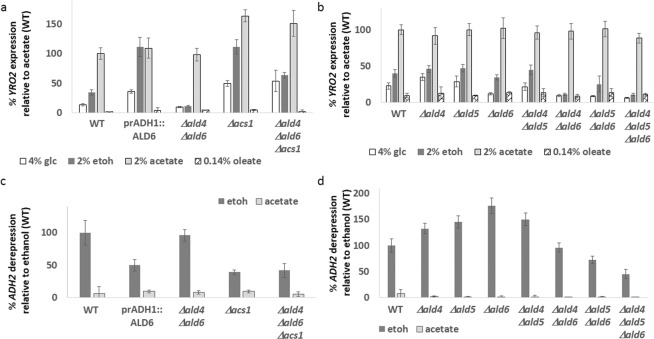
(**a**,**b**) Cells were grown for 24 hours in 5 ml of the indicated media (without dilution) and *YRO2* expression measured. Expression is normalized against WT in acetate medium. N = 3. Error bars are one standard deviation. (**c**,**d**) Cells were grown overnight in 4% glucose. In the morning, cells were diluted and grown for an additional 3 hours in 4% glucose media. After measuring the basal *ADH2* expression (t = 0), cells were washed three times with DDW and resuspended in media containing either ethanol (2%) or sodium acetate pH6 (2%). *ADH2* expression was determined after 3 hours. Rate of expression is normalized against WT cells in ethanol media. N = 3. Error bars are one standard deviation.

If acetate synthesized intracellularly by ethanol catabolism can be sensed to cause *YRO2* expression, then we would expect genetic manipulation of this pathway to exert effects upon gene expression (Fig. 4). Indeed, overexpression of *ALD6* results in a dramatic increase in *YRO2* expression when cells are grown on glucose or ethanol, but not when grown on acetate or oleate, suggestive of increased acetate production from acetyladehyde. Similarly, *YRO2* expression was reduced in glucose- or ethanol-containing media in *Δald4 Δald6* cells, which lack two of the five redundant enzymes that convert acetylaldehyde into acetate (*YRO2* expression in response to acetate was unchanged in *Δald4Δald6* cells.) *YRO2* expression in glucose or ethanol was restored in *Δald4Δald6Δacs1* cells (Fig. 5a). We compared the contribution of *ALD4, ALD5* and *ALD6*. Single deletions alone did not affect *YRO2* expression, nor did the *Δald4Δald5* double deletion. Deletion of *ALD6* in combination with either *Δald4* or *Δald5* dramatically reduced *YRO2* expression in glucose or ethanol media, and a triple *Δald4Δald5Δald6* strain eliminates *YRO2* expression to the background levels observed in oleic acid (Fig. 5b).

Mutations that increase acetate production (*ALD6* overexpression) or reduce its consumption (*Δacs1*) lower *ADH2* expression levels (Fig. 5c). *ADH2* expression was elevated in *Δald6* cells when cells were grown on 2% ethanol (Fig. 5d), but progressively declined upon further deletion of *ALD* genes. As expected, *ADH2* expression remained low when cells were grown in acetate medium. We suspect that this is due to another layer of regulation of *ADH2* expression by the very toxic intermediate^52^ acetylaldehyde induced by the restriction of acetyladehyde outflow to acetate.

## Discussion

Carbon catabolite repression is often considered to be a matter of “good” carbon sources, such as glucose and fructose, actively preventing the metabolism of other carbon sources, and has been much investigated. However, the relationships between poor carbon sources has been of scant enquiry, and thus it was unclear whether similar inter-sugar relationships exist for them, similarly to the relation between glucose and other sugars. Here we have shown that part of the acetate response involves downregulating of *ADH2* expression. We also show that there is no positive signal needed to promote *ADH2* expression; rather, its expression is negatively regulated by glucose (via Snf1 inhibition of Adr1)^44^, by galactose^53^ and its downstream product acetate via the Haa1 transcription factor acting as a repressor (Fig. 4). Similarly to glucose, acetate represses *ADH2* expression even when other carbon sources are present. Although gene expression in response to acetate has been previously investigated^28,29^, this level of regulation has been overlooked since glucose independently inhibits *ADH2* expression. In addition, most studies involving acetate provide acetic acid as a non-metabolizable (due to glucose) stressor (pH 4), with both acid and acetate contributing to the stress response. Here we have provided dissociated acetate (pH6) as the sole carbon source. Extracellular acetate (pH 6) or intracellularly produced acetate produced via catabolism of glucose or ethanol provoked the Haa1 dependent response previously demonstrated for acetic acid^50,51^, thus showing that the response is not due to protons but rather is due to the acetate moiety. The intracellular acetate sensor remains to be identified.

We found that ADH activity attributable to *ADH2* correlates with *ADH2* expression in cells grown with ethanol or acetate as their sole carbon source, although all ADH activity was attenuated in oleic acid grown cells, suggesting further post-transcriptional regulation to occur. It is possible that signaling leading to post translational modification and inhibition of the ADH enzymes may arise from the cleavage and processing of Mga2/Spt23^54^, or as a consequence of changes to membrane fluid dynamics activating sensors such as Mid1 and Mid2^55,56^. Although oleic acid does not affect *ADH2* or *YRO2* expression, it is probable that other inhibitory relationships exist between other carbon sources, which in most studies are masked by an overall glucose repression. However, under conditions where glucose becomes limiting, such mechanisms of metabolic repression are likely to be of significance.

We then utilized the gene expression of *ADH2* and *YRO2* to determine whether acetate is sensed extracelullarly, like glucose or osmotic stress, or whether acetate needs to enter the cell in order to elicit a response. Deletion of all three acetate transporters was required to restore *ADH2* expression in acetate, and Haa1-GFP did not localize to the nucleus, nor was *YRO2* expressed, in strains lacking these transporters when exposed to acetate, although slowing the efflux of acetate to acetyl-coA by deletion of *ACS1* did partially restore *YRO2* expression.

Since acetate is sensed intracellularly, we reasoned that metabolically produced acetate would also elicit an acetate response, albeit to a lesser degree than upon growth with acetate as the sole carbon source. Indeed, the expression of *ADH2* or *YRO2* was unaffected in a strain deleted for the acetate transporters when cells were grown on either glucose or ethanol as their sole carbon source, further confirming that the effects in acetate media of these deletions is due to a lack of acetate uptake. We further found that mutations in the metabolic pathway that increase acetate production or lower its utilization increase the acetate response, whereas mutations that lower conversion of acetylaldehyde to acetate lower the acetate response, the major enzyme responsible being *ALD6*.

Strikingly, all the metabolites involved in this pathway are toxic^21,22,52,57,58^, and yet acetate is produced faster than it is consumed (thus leading to the acetate response in glucose and ethanol grown cells). However, the consequences of upregulating acetyl-coA production may not be benign, as cytoplasmic acetyl-coA is a regulator of autophagy^59^ and directly correlates to histone acetylation levels^60^. Therefore the yeast response seems to be not to promote acetate metabolism, but rather to deal with the resultant stress^27,49,61,62^, and as shown here, to reduce expression of *ADH2*. Together, our results uncover a mechanism by which acetate inhibits its own production, resulting in homeostasis of acetate levels (Fig. 4).

## Materials and Methods

Strains used are listed in Table S1**;** plasmids used are listed in Table S2. All strains are related to BY4741^63^ except for W303-1b and V5. Standard yeast molecular biology techniques were used for yeast manipulations. To overexpress *ALD6*, ALD6-GFP was inserted into ycpADH1^64^ digested with SalI/SpeI together with the Cyc1 terminator from pUG34. To make the ACS2-LacZ reporter plasmid, the *ZWF1* promoter was excised from a plasmid containing *prZWF1::*LacZ in Ycp50^65^ by BamHI/HindIII digestion, and replaced with 1000 bp of the *ACS2* promoter by gap repair.

Media was prepared with 8 g/litre YNB, with 0.0286 g/liter adeneine, tryptophan, histidine, arginine, methionine, 0.0714 g/liter phenylalanine, 0.0857 g/liter tyrosine, lysine, 0.114 g/liter isoleucine, 0.143 g/liter glutamate, aspartate, 0.214 g/liter valine, 0.286 g/liter threonine, 0.571 g/liter serine to make synthetic –LU media. Standard carbon source concentrations were 4% for glucose, 2% for ethanol, 3% for glycerol, 2% for acetate (as sodium acetate at pH6), and 0.14% oleate (in 1% tween-80)^66^. The pH of the resultant media were 4.7 for glucose, 4.7 for ethanol, 4.5 for oleate and 6 for acetate. (In contrast, glucose containing media with 60 mM acetic acid^51^ has a pH of 3.9.) The pH of the media after 24 h of yeast growth was 3.5 for glucose, 4.7 for ethanol, 4.5 for oleate and 6 for acetate. Ethanol containing medium was buffered with potassium phosphate at pH6 where indicated in Fig. 1a.

For *ADH2* induction assays, cells were grown in 4% glucose overnight to ensure complete repression of ADH2 expression, diluted in the morning and grown for an additional 3 hours (t = 0), washed 3x with water, and resuspended in media containing indicated carbon sources for a further 3 hours (t = 3). The data presented is the t = 3 − t = 0/3 and normalized to the WT ethanol sample from that experiment. Typical *ADH2* expression was 1500 Miller Units per hour. For *YRO2* and other gene expression assays, cells were taken from glucose plates and grown in 5 ml of indicated media for 24 hours without dilution, to prevent loss of secreted metabolites from the medium. Cells visibly grew during this period.

### β-galactosidase assays

β-galactosidase assays were performed using log phase cells. Cell concentration was determined by reading 80 μl of cells at 595 nm. 20 μl of cells were added to the β-galactosidase reaction mix (40 μl YPER (Pierce 78990), 80 μl Z-buffer (120 mM Na_2_HPO_4_, 80 mM NaH_2_PO_4_, 20 mM KCl, 2 mM MgSO_4_), 24 μl ONPG (4 mg/ml), 0.4 μl β-mercaptoethanol) and incubated at 30 °C for 10 minutes for *ADH2*, 25 minutes for *YRO2* and for 15 minutes for other genes. Reactions were stopped by addition of 56 μl 1 M Na_2_CO_3_. The eppendorf tubes were centrifuged for 1 minute at full-speed to pellet the cell debris, and 200 μl supernatant was removed and absorbance read at 415 nm using a microplate reader. Miller Units were calculated by the equation Miller Units = (1000*A_415_)/(time*0.02*A_595_ − 0.055, where the A_415_ and A_595_ has been corrected for blanking and path length (final path length = 1 cm). Three biological replicates were measured. Error bars are ± 1 standard deviation.

### Microscopy

5 μl of log phase cells were imaged using an EVOS microscope (60x objective) with the GFP filter for GFP and the Texas Red filter for Cherry. The dimensions of each panel corresponds to 20 μm × 20 μm. Cells were not concentrated before imaging, to prevent perturbation to their environment^67^. Images were processed using the brightness/contrast function of Image J, to give a black background. For statistics, over 200 cells were counted. Experiments were repeated at least three times on different days. All figures shown in the manuscript are of identical magnification, a size bar is provided in Fig. 3a.

### ADH assays

Cells were grown overnight in their respective media. 0.8OD_600_ was harvested and vortexed with glass beads for 20 minutes at 4 °C with 100 μl PBS + 0.1% Triton X-100 with Protease (Roche) and Phosphatase (Pierce) inhibitors. Supernatant was centrifuged at 4 °C for 10 minutes at 13000 g. An ADH assay kit (Sigma MAK053) was used to determine ADH activity – all volumes were halved from the values suggested in the datasheet, and the amount of developer reduced by half again. Reactions were rapid and completed within 5 minutes. An *adh2*Δ strain was included as a control. ADH activity is shown as milliUnits per OD_600_ of yeast.

## Supplementary information

Suppl. Information

## References

[1] Tatebayashi K (2007). Transmembrane mucins Hkr1 and Msb2 are putative osmosensors in the SHO1 branch of yeast HOG pathway. The EMBO journal.

[2] Sabina J, Johnston M (2009). Asymmetric signal transduction through paralogs that comprise a genetic switch for sugar sensing in Saccharomyces cerevisiae. The Journal of biological chemistry.

[3] Panchaud N, Peli-Gulli MP, De Virgilio C (2013). Amino acid deprivation inhibits TORC1 through a GTPase-activating protein complex for the Rag family GTPase Gtr1. Sci Signal.

[4] Okazaki S, Naganuma A, Kuge S (2005). Peroxiredoxin-mediated redox regulation of the nuclear localization of Yap1, a transcription factor in budding yeast. Antioxid Redox Signal.

[5] Simpson-Lavy K, Kupiec M (2018). A reversible liquid drop aggregation controls glucose response in yeast. Current genetics.

[6] Gancedo JM (1998). Yeast carbon catabolite repression. Microbiol Mol Biol Rev.

[7] Soontorngun N (2017). Reprogramming of nonfermentative metabolism by stress-responsive transcription factors in the yeast Saccharomyces cerevisiae. Current genetics.

[8] Pascual-Ahuir A, Manzanares-Estreder S, Timon-Gomez A, Proft M (2018). Ask yeast how to burn your fats: lessons learned from the metabolic adaptation to salt stress. Current genetics.

[9] Adnan Muhammad, Zheng Wenhui, Islam Waqar, Arif Muhammad, Abubakar Yakubu, Wang Zonghua, Lu Guodong (2017). Carbon Catabolite Repression in Filamentous Fungi. International Journal of Molecular Sciences.

[10] Alam, M. A. & Kelly, J. M. Proteins interacting with CreA and CreB in the carbon catabolite repression network in Aspergillus nidulans. *Current genetics* **63**, 669–683. 10.1007/s00294-016-0667-2 27915380

[11] Matar Khalid Abdelkarim Omer, Chen Xiaofeng, Chen Dongjie, Anjago Wilfred Mabeche, Norvienyeku Justice, Lin Yahong, Chen Meilian, Wang Zonghua, Ebbole Daniel J., Lu Guo-dong (2016). WD40-repeat protein MoCreC is essential for carbon repression and is involved in conidiation, growth and pathogenicity of Magnaporthe oryzae. Current Genetics.

[12] Kremling A, Geiselmann J, Ropers D, de Jong H (2015). Understanding carbon catabolite repression in Escherichia coli using quantitative models. Trends Microbiol.

[13] Romano A (2017). Fats for thoughts: An update on brain fatty acid metabolism. Int J Biochem Cell Biol.

[14] Sanz P, Viana R, Garcia-Gimeno MA (2016). AMPK in Yeast: The SNF1 (Sucrose Non-fermenting 1) Protein Kinase Complex. Exs.

[15] Kim JH, Roy A, Jouandot D, Cho KH (2013). The glucose signaling network in yeast. Biochimica et biophysica acta.

[16] Zhang N, Cao L (2017). Starvation signals in yeast are integrated to coordinate metabolic reprogramming and stress response to ensure longevity. Current genetics.

[17] Paiva S (2009). Glucose-induced ubiquitylation and endocytosis of the yeast Jen1 transporter: role of lysine 63-linked ubiquitin chains. The Journal of biological chemistry.

[18] Vilela-Moura A (2011). The impact of acetate metabolism on yeast fermentative performance and wine quality: reduction of volatile acidity of grape musts and wines. Appl Microbiol Biotechnol.

[19] Styger G, Prior B, Bauer FF (2011). Wine flavor and aroma. J Ind Microbiol Biotechnol.

[20] Heit C, Martin SJ, Yang F, Inglis DL (2018). Osmoadaptation of wine yeast (Saccharomyces cerevisiae) during Icewine fermentation leads to high levels of acetic acid. J Appl Microbiol.

[21] Giannattasio S, Guaragnella N, Zdralevic M, Marra E (2013). Molecular mechanisms of Saccharomyces cerevisiae stress adaptation and programmed cell death in response to acetic acid. Front Microbiol.

[22] Casatta N, Porro A, Orlandi I, Brambilla L, Vai M (2013). Lack of Sir2 increases acetate consumption and decreases extracellular pro-aging factors. Biochimica et biophysica acta.

[23] Piper PW (2011). Resistance of yeasts to weak organic acid food preservatives. Adv Appl Microbiol.

[24] Maiorella B, Blanch HW, Wilke CR (1983). By-product inhibition effects on ethanolic fermentation by Saccharomyces cerevisiae. Biotechnol Bioeng.

[25] Palmqvist E, Grage H, Meinander NQ, Hahn-Hagerdal B (1999). Main and interaction effects of acetic acid, furfural, and p-hydroxybenzoic acid on growth and ethanol productivity of yeasts. Biotechnol Bioeng.

[26] Sanchez I Nogue V, Narayanan V, Gorwa-Grauslund MF (2013). Short-term adaptation improves the fermentation performance of Saccharomyces cerevisiae in the presence of acetic acid at low pH. Appl Microbiol Biotechnol.

[27] Cunha JT (2018). HAA1 and PRS3 overexpression boosts yeast tolerance towards acetic acid improving xylose or glucose consumption: unravelling the underlying mechanisms. Appl Microbiol Biotechnol.

[28] Mira NP, Becker JD, Sa-Correia I (2010). Genomic expression program involving the Haa1p-regulon in Saccharomyces cerevisiae response to acetic acid. OMICS.

[29] Mira NP (2011). Identification of a DNA-binding site for the transcription factor Haa1, required for Saccharomyces cerevisiae response to acetic acid stress. Nucleic Acids Res.

[30] Paiva S, Devaux F, Barbosa S, Jacq C, Casal M (2004). Ady2p is essential for the acetate permease activity in the yeast Saccharomyces cerevisiae. Yeast.

[31] Casal M, Paiva S, Andrade RP, Gancedo C, Leao C (1999). The lactate-proton symport of Saccharomyces cerevisiae is encoded by JEN1. J Bacteriol.

[32] Casal M, Paiva S, Queiros O, Soares-Silva I (2008). Transport of carboxylic acids in yeasts. FEMS Microbiol Rev.

[33] Mollapour M, Piper PW (2006). Hog1p mitogen-activated protein kinase determines acetic acid resistance in Saccharomyces cerevisiae. FEMS Yeast Res.

[34] de Smidt O, du Preez JC, Albertyn J (2012). Molecular and physiological aspects of alcohol dehydrogenases in the ethanol metabolism of Saccharomyces cerevisiae. FEMS Yeast Res.

[35] Ganzhorn AJ, Green DW, Hershey AD, Gould RM, Plapp BV (1987). Kinetic characterization of yeast alcohol dehydrogenases. Amino acid residue 294 and substrate specificity. The Journal of biological chemistry.

[36] Bakker BM (2000). The mitochondrial alcohol dehydrogenase Adh3p is involved in a redox shuttle in Saccharomyces cerevisiae. J Bacteriol.

[37] Boubekeur S, Camougrand N, Bunoust O, Rigoulet M, Guerin B (2001). Participation of acetaldehyde dehydrogenases in ethanol and pyruvate metabolism of the yeast Saccharomyces cerevisiae. European journal of biochemistry.

[38] Saint-Prix F, Bonquist L, Dequin S (2004). Functional analysis of the ALD gene family of Saccharomyces cerevisiae during anaerobic growth on glucose: the NADP+−dependent Ald6p and Ald5p isoforms play a major role in acetate formation. Microbiology.

[39] Kratzer S, Schuller HJ (1995). Carbon source-dependent regulation of the acetyl-coenzyme A synthetase-encoding gene ACS1 from Saccharomyces cerevisiae. Gene.

[40] Van den Berg MA, Steensma HY (1995). ACS2, a Saccharomyces cerevisiae gene encoding acetyl-coenzyme A synthetase, essential for growth on glucose. European journal of biochemistry.

[41] Zhang M, Galdieri L, Vancura A (2013). The yeast AMPK homolog SNF1 regulates acetyl coenzyme A homeostasis and histone acetylation. Mol Cell Biol.

[42] Galdieri L, Zhang T, Rogerson D, Lleshi R, Vancura A (2014). Protein acetylation and acetyl coenzyme a metabolism in budding yeast. Eukaryot Cell.

[43] Strijbis K, Distel B (2010). Intracellular acetyl unit transport in fungal carbon metabolism. Eukaryot Cell.

[44] Ratnakumar Sooraj, Kacherovsky Nataly, Arms Erin, Young Elton T. (2009). Snf1 Controls the Activity of Adr1 Through Dephosphorylation of Ser230. Genetics.

[45] Irani M, Taylor WE, Young ET (1987). Transcription of the ADH2 gene in Saccharomyces cerevisiae is limited by positive factors that bind competitively to its intact promoter region on multicopy plasmids. Mol Cell Biol.

[46] Ciriacy M (1976). Cis-dominant regulatory mutations affecting the formation of glucose-repressible alcohol dehydrogenase (ADHII) in Saccharomyces cerevisiae. Mol Gen Genet.

[47] Wills C, Martin T (1984). Extracellular conditions affecting the induction of yeast alcohol dehydrogenase II. Biochimica et biophysica acta.

[48] Young MJ, Court DA (2008). Effects of the S288c genetic background and common auxotrophic markers on mitochondrial DNA function in Saccharomyces cerevisiae. Yeast.

[49] Fernandes AR, Mira NP, Vargas RC, Canelhas I, Sa-Correia I (2005). Saccharomyces cerevisiae adaptation to weak acids involves the transcription factor Haa1p and Haa1p-regulated genes. Biochemical and biophysical research communications.

[50] Sugiyama M (2014). Nuclear localization of Haa1, which is linked to its phosphorylation status, mediates lactic acid tolerance in Saccharomyces cerevisiae. Applied and environmental microbiology.

[51] Collins, M. E., Black, J. J. & Liu, Z. Casein Kinase I Isoform Hrr25 Is a Negative Regulator of Haa1 in the Weak Acid Stress Response Pathway in Saccharomyces cerevisiae. *Applied and environmental microbiology* **83**, 10.1128/AEM.00672-17 (2017). 10.1128/AEM.00672-17 PMC5478977 28432100

[52] Matsufuji Y (2008). Acetaldehyde tolerance in Saccharomyces cerevisiae involves the pentose phosphate pathway and oleic acid biosynthesis. Yeast.

[53] Simpson-Lavy K, Xu T, Johnston M, Kupiec M (2017). The Std1 Activator of the Snf1/AMPK Kinase Controls Glucose Response in Yeast by a Regulated Protein Aggregation. Mol Cell.

[54] Ballweg S, Ernst R (2017). Control of membrane fluidity: the OLE pathway in focus. Biol Chem.

[55] Mishra R (2017). Protein kinase C and calcineurin cooperatively mediate cell survival under compressive mechanical stress. Proc Natl Acad Sci USA.

[56] Ozeki-Miyawaki C, Moriya Y, Tatsumi H, Iida H, Sokabe M (2005). Identification of functional domains of Mid1, a stretch-activated channel component, necessary for localization to the plasma membrane and Ca2+ permeation. Exp Cell Res.

[57] Hu J (2014). Tor-Sch9 deficiency activates catabolism of the ketone body-like acetic acid to promote trehalose accumulation and longevity. Aging Cell.

[58] Kubota S (2004). Effect of ethanol on cell growth of budding yeast: genes that are important for cell growth in the presence of ethanol. Biosci Biotechnol Biochem.

[59] Eisenberg T (2014). Nucleocytosolic depletion of the energy metabolite acetyl-coenzyme a stimulates autophagy and prolongs lifespan. Cell Metab.

[60] Galdieri L, Vancura A (2012). Acetyl-CoA carboxylase regulates global histone acetylation. The Journal of biological chemistry.

[61] Tani M, Funato K (2018). Protection mechanisms against aberrant metabolism of sphingolipids in budding yeast. Current genetics.

[62] Singh P, Li R (2018). Emerging roles for sphingolipids in cellular aging. Current genetics.

[63] Brachmann CB (1998). Designer deletion strains derived from Saccharomyces cerevisiae S288C: a useful set of strains and plasmids for PCR-mediated gene disruption and other applications. Yeast.

[64] Pedruzzi I, Burckert N, Egger P, De Virgilio C (2000). Saccharomyces cerevisiae Ras/cAMP pathway controls post-diauxic shift element-dependent transcription through the zinc finger protein Gis1. The EMBO journal.

[65] Karpov DS, Spasskaya DS, Tutyaeva VV, Mironov AS, Karpov VL (2013). Proteasome inhibition enhances resistance to DNA damage via upregulation of Rpn4-dependent DNA repair genes. FEBS letters.

[66] Fan Q (2018). Rad5 coordinates translesion DNA synthesis pathway by recognizing specific DNA structures in saccharomyces cerevisiae. Current genetics.

[67] Nossmann M, Pieper J, Hillmann F, Brakhage AA, Munder T (2018). Generation of an arginine-tRNA-adapted Saccharomyces cerevisiae strain for effective heterologous protein expression. Current genetics.

