# Supplementary material for: Carbon Catabolite Repression in Yeast is Not Limited to Glucose
Source: Sci Rep. 2019 Apr 24;9:6491. doi: 10.1038/s41598-019-43032-w (PMC6482301; doi:10.1038/s41598-019-43032-w)
Supplement: Supplementary file 1 — Suppl. Information [file 41598_2019_43032_MOESM1_ESM.pdf]

Supplementary Material for:

Carbon Catabolite Repression in Yeast is Not Limited to Glucose

Kobi Simpson-Lavy and Martin Kupiec[1]

School of Molecular Cell Biology & Biotechnology, Tel Aviv University, Ramat Aviv 69978, Israel.

[1.] Corresponding author: martin@post.tau.ac.il

Table S1. Yeast Strains used in this study.

All yeast strains are derived from BY4741 *MATa his3Δ1, leu2Δ0, ura3Δ0, met15Δ0*.

Number	Genotype	Source
1	WT	Lab collection
2	*haa1::KAN*	Deletion collection
3	*hrr25::KAN*	Liu Z (1)
4	*hrr25*Δ::*kanMX4* [pRS415-HRR25(E52D)]	Liu Z (1)
5	*hrr25*Δ::*kanMX4 haa1*::*HIS3* [pRS415-HRR25(E52D)]	Liu Z (1)
6	*msn5::NAT*	This study
7	*haa1::KAN msn5::NAT*	This study
8	*adr1::KAN*	Deletion collection
9	*adr1::KAN haa1::HygB*	This study
10	*ady2::KAN*	Deletion collection
11	*fps1::KAN*	Deletion collection
12	*fps1::KAN ady2::HygB*	This study
13	*fps1::KAN jen1::NAT*	This study
14	*jen1::NAT*	This study
15	*ady2::KAN jen1::NAT*	This study
16	*fps1::KAN jen1::NAT ady2::HygB*	This study
17	*acs1::KAN*	Deletion collection
18	*fps1::KAN jen1::NAT ady2::HygB acs1::HIS3*	This study
19	*ald4::KAN*	Deletion collection
20	*ald5:KAN*	Deletion collection
21	*ald6::KAN*	Deletion collection
22	*ald4::KAN ald5::NAT*	This study
23	*ald4::KAN ald6::NAT*	This study
24	*ald6::KAN ald5::NAT*	This study
25	*ald4::KAN ald5::HygB ald6::NAT*	This study
26	*ald4::KAN ald6::NAT acs1::HIS3*	This study
27	*adh2*::KAN	Deletion collection

Table S2. Plasmids Used in this study

Name	Genotype	Vector/Markers	Source
prs315		LEU2	Lab collection
pBGM18	pr*ADH2*::LacZ	pRS316, 313 (changed by gap repair)	Elton Young (2)
YRO2-LacZ	*1929bp prYRO2*::LacZ	pRS416 URA3	Z. Liu (1)
Haa1-GFP	pr*HAA1::HAA1-GFP*	pRS416 URA3	Z. Liu (1)
Adr1^{S230A}	pr*ADH1::ADR1*^{S230A}	CEN KAN LEU2 (changed from TRP1)	Elton Young (3)

ALD6-OE	pr*ADH1::ALD6-GFP*	pRS415 LEU2	This study
ALD4-LacZ	pr*ALD4::*LacZ	yep357 2μ URA3	J. Ariño (4)
ALD5-LacZ	pr*ALD5::*LacZ	yep357 2μ URA3	J. Ariño (4)
ALD6-LacZ	pr*ALD6::*LacZ	yep357 2μ URA3	J. Ariño (4)
ACS1-LacZ	pr*ACS*1::LacZ	yep357 2μ URA3	H. Schueller (5)
ACS2-LacZ	1000bp pr*ACS*2::LacZ	Ycp50 CEN URA3	This study

1. Collins ME, Black JJ, & Liu Z (2017) Casein Kinase I Isoform Hrr25 Is a Negative Regulator of Haa1 in the Weak Acid Stress Response Pathway in Saccharomyces cerevisiae. *Appl Environ Microbiol* 83(13).
2. Dombek KM, Voronkova V, Raney A, & Young ET (1999) Functional analysis of the yeast Glc7-binding protein Reg1 identifies a protein phosphatase type 1-binding motif as essential for repression of ADH2 expression. (Translated from eng) *Mol Cell Biol* 19(9):6029-6040 (in eng).
3. Ratnakumar S, Kacherovsky N, Arms E, & Young ET (2009) Snf1 controls the activity of adr1 through dephosphorylation of Ser230. (Translated from eng) *Genetics* 182(3):735-745 (in eng).
4. Ruiz A, Serrano R, & Arino J (2008) Direct regulation of genes involved in glucose utilization by the calcium/calcineurin pathway. *J Biol Chem* 283(20):13923-13933.
5. Kratzer S & Schuller HJ (1995) Carbon source-dependent regulation of the acetyl-coenzyme A synthetase-encoding gene ACS1 from Saccharomyces cerevisiae. *Gene* 161(1):75-79.

Suppl. Figure legends

Figure S1: Simplified diagram illustrating the metabolic pathways leading to cytoplasmic acetyl-coA, together with the carbon sources used for this work. Resp. = respiration conditions. Cyto. = cytoplasm Mito. = mitochondrial.

Figure S2 a, b, c, d, e. Cells were grown for 24 hours in 5ml of the indicated media (without dilution) and indicated gene expression measured. Expression is normalized against WT in ethanol medium. N=3. Error bars are one standard deviation.

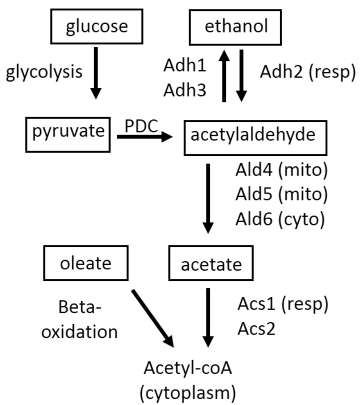

glucose
glycolysis
ethanol
Adh1
Adh3
Adh2 (resp)
pyruvate
PDC
acetylaldehyde
Ald4 (mito)
Ald5 (mito)
Ald6 (cyto)
oleate
acetate
Beta-
oxidation
Acs1 (resp)
Acs2
Acetyl-coA
(cytoplasm)

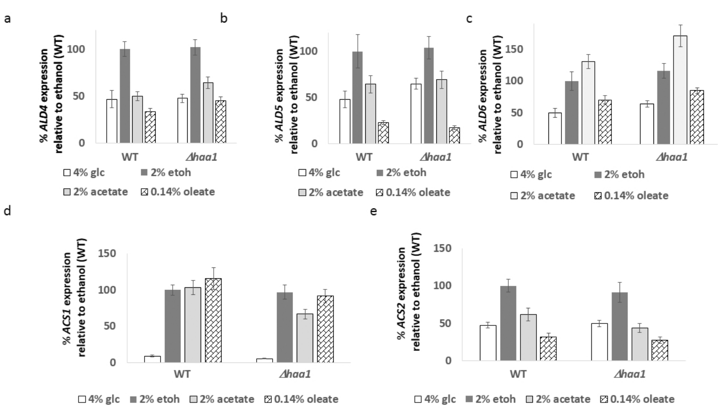
a
% ALD4 expression relative to ethanol (WT)
100
50
0
WT
Δhaa1
4% glc
2% etoh
2% acetate
0.14% oleate
b
% ALD5 expression relative to ethanol (WT)
100
50
0
WT
Δhaa1
4% glc
2% etoh
2% acetate
0.14% oleate
c
% ALD6 expression relative to ethanol (WT)
150
100
50
0
WT
Δhaa1
4% glc
2% etoh
2% acetate
0.14% oleate
d
% ACS1 expression relative to ethanol (WT)
150
100
50
0
WT
Δhaa1
4% glc
2% etoh
2% acetate
0.14% oleate
e
% ACS2 expression relative to ethanol (WT)
150
100
50
0
WT
Δhaa1
4% glc
2% etoh
2% acetate
0.14% oleate
